# Fibrin Gel as a Versatile Biomaterial Platform in the Biomedical Landscape: Chemical, Physical, and Biological Insights

**DOI:** 10.3390/gels12050351

**Published:** 2026-04-22

**Authors:** Sabrina Caria, Jessica Petiti, Gerardina Ruocco, Lorenzo Mino, Raffaella Romeo, Gabriele Viada, Laura Revel, Federico Picollo, Valeria Chiono, Carla Divieto

**Affiliations:** 1 Physics Department, Università di Torino, 10125 Turin, Italy; federico.picollo@unito.it; 2 Division of Advanced Materials Metrology and Life Sciences, Istituto Nazionale di Ricerca Metrologica (INRIM), 10135 Turin, Italy; j.petiti@inrim.it (J.P.); l.revel@inrim.it (L.R.); c.divieto@inrim.it (C.D.); 3 Department of Mechanical and Aerospace Engineering, Politecnico di Torino, 10129 Turin, Italy; gerardina.ruocco@polito.it (G.R.); valeria.chiono@polito.it (V.C.); 4 POLITO Biomedlab, Politecnico di Torino, 10129 Turin, Italy; 5 Interuniversity Center for the Promotion of the 3Rs Principles in Teaching and Research, 10129 Turin, Italy; 6 Department of Chemistry and Interdepartmental Centre NIS, Università di Torino, 10125 Turin, Italy; lorenzo.mino@unito.it; 7 Division of Applied Metrology and Engineering, Istituto Nazionale di Ricerca Metrologica (INRIM), 10135 Turin, Italy; r.romeo@inrim.it; 8 Department of Chemistry, NIS Interdepartmental Centre and INSTM Reference Centre, Università di Torino, 10135 Turin, Italy; gabriele.viada@unito.it; 9 INFN Turin Section, 10125 Turin, Italy

**Keywords:** fibrin gel, protein-based polymer, hydrogel characterization, fibrin gel properties, cytocompatibility, physicochemical properties

## Abstract

Fibrin gel, a protein-based polymer naturally generated during coagulation, has garnered attention in the biomedical field for applications such as fibrin glue, due to its specific physical and biological properties. Despite it, low mechanical strength and rapid degradation limited its utilization for biomedical applications. This study presents a reproducible protocol for the synthesis of pure fibrin hydrogels, aimed at achieving predictable structural properties through the precise calibration of fibrinogen and thrombin concentrations. By examining the mechanical and morphological characteristics, as well as the relationship between reagent concentrations and structural integrity, this research assesses impacts on swelling behavior, water absorption, and overall stability. Through a comprehensive analytical approach, we identified an optimal formulation, specifically 2.25 mg/mL fibrinogen and 1.375 U/mL thrombin, that effectively balances structural integrity with high cytocompatibility. The results demonstrate that this calibrated approach ensures high procedural reproducibility and a well-defined hydrogel architecture without the need for exogenous chemical cross-linkers. This work provides a robust methodological framework to overcome the common lack of reproducibility in fibrin-based hydrogel studies, positioning these materials as highly reliable candidates for advanced 3D in vitro models and biomedical applications.

## 1. Introduction

The natural tissue environment (NTE) is a complex 3D microenvironment that surrounds cells within living tissues. It comprises the extracellular matrix, soluble molecules such as growth factors, and other cellular components that provide structural support and biochemical cues for cell survival, function, and organization [1]. Mimicking the NTE in vitro models is crucial because it recreates the physiological microenvironment that regulates cell behavior, leading to more authentic cellular responses and more predictive models for research. By replicating the complexities of native tissues, these advanced models provide a more accurate basis for studying disease progression, understanding how tissues function and regenerate, and developing and testing new drugs and therapies [2,3]. Existing models designed to mimic NTE can be broadly gathered into three groups: 2D in vitro models, 3D in vitro models, and in vivo animal models.

To overcome the limitations of 2D in vitro models, including the lack of 3D tissue organization and reduced predictive value for patient-specific responses [3,4] and of in vivo animal models, including ethical concerns, long and expensive experiments, and physiological differences with humans [5], 3D in vitro models have emerged as viable alternatives, capable of better mimicking in vivo conditions and improving the predictability of clinical outcomes and toxicity.

Recent advancements in biomedicine, materials science, and bioengineering have facilitated the development of various 3D in vitro models, which have shown superior predictive power regarding disease progression and therapeutic efficacy compared to conventional models, indicating a promising future across multiple fields in healthcare and research [6]. Examples of 3D in vitro models include organoids, which are self-organized miniature aggregates of cells; scaffold-based models, where cells are cultured within biomaterials that simulate the extracellular matrix; microfluidic 3D models, for example, organ-on-a-chip devices featuring perfusable channels; and 3D bioprinted models, in which cells and bioinks are assembled into intricate 3D structures [7,8]. Biomaterials are essential elements of 3D in vitro models, offering a 3D platform that replicates the extracellular matrix to facilitate cell growth and behavior [9]. Among the various classes of biomaterials, hydrogels are extensively researched and employed in biomedical applications due to the distinctive properties that enable them to effectively replicate NTEs.

Indeed, hydrogels represent a unique class of materials characterized by their 3D structures, formed through the cross-linking of water-soluble polymers. This process results in a biphasic material that combines a porous, permeable solids network with at least 10% water or other interstitial fluids. The solid component consists of a water-insoluble polymeric framework capable of retaining significant amounts of water or biological fluids while maintaining its structural integrity, a property further enhanced by the stability and durability provided by the cross-linking process. Due to their exceptional water absorption and retention capabilities, hydrogels are particularly valuable in a variety of applications, especially within the biomedical field, where they can effectively replicate the aqueous environments of animal tissues and support the physiological functions of cells [10].

Hydrogels can be broadly categorized into two main types based on the origin of their components: natural and synthetic [11]. Natural polymers are characterized by their high biocompatibility, biodegradability, accessibility, stability, non-toxicity, and cost-effectiveness [12]. These inherent advantages become particularly evident at the end of their life cycle, as the residues can be reintegrated into the environment in a more sustainable manner, ultimately serving as a source of organic matter [13]. Among the various types of natural polymers, proteins and polysaccharides are especially valuable due to their affordability, abundance, biodegradability, and biocompatibility [13].

Among protein-based hydrogels, fibrin-based hydrogels are particularly interesting due to their ability to closely mimic the extracellular matrix and effectively promote cell adhesion, migration, and wound healing. Additionally, their natural hemostatic capabilities have facilitated their extensive clinical use in surgery and tissue engineering, spanning a wide range of applications in a regenerative medicine context [14,15,16].

Fibrin is a naturally occurring protein in the human body that plays a crucial role in the coagulation process. It is essential for the formation of blood clots, creating a mesh-like structure that captures platelets and red blood cells, thereby effectively sealing injured blood vessels and stopping bleeding [14]. The formation of fibrin occurs via a polycondensation reaction involving fibrinogen as the primary substrate, thrombin as the catalyst, sodium ions (Na^+^), which enhance the catalyst activity, and calcium ions (Ca^2+^), which activate additional coagulation factors, allowing for the stabilization of the 3D network [17]. When blood vessels are injured, prothrombin is converted to thrombin via proteolytic reaction catalyzed by prothrombinase. Once thrombin is generated, Na^+^ bind to specific sites, further enhancing its catalytic properties [18]. Activated-thrombin then interacts with fibrinogen, resulting in the release of fibrinopeptides A and B from the α and β chains. This cleavage produces fibrin monomers that spontaneously polymerize to form a fibrin clot [19]. After polymerization, coagulation factor XIIIa and Ca^2+^ promote the covalent cross-linking of the fibrin network, reinforcing the clot and protecting it from mechanical or enzymatic disruption [20]. While thrombin converts soluble fibrinogen into fibrin monomers that aggregate spontaneously, fibrin monomers are only held together by weak hydrogen bonds. Ca^2+^-activated factor XIIIa provides the covalent stabilization required for structural integrity [21].

In recent years, fibrin-based materials, such as fibrin glue and sealants, have found several applications in the biomedical field due to their ability to fulfill specific physical and biological requirements [22]. Despite its widespread use, the clinical and bioengineering potential of fibrin is often constrained by several inherent drawbacks. The most significant challenges include its limited mechanical stiffness and a tendency toward rapid, unpredictable degradation when exposed to cellular activities [23]. These factors frequently lead to scaffold contraction, which compromises the structural integrity and shape fidelity required for applications in tissue engineering [24,25,26,27] and 3D bioprinting [28,29]. To address these limitations, recent studies on fibrin gels have tried to overcome the inherent mechanical weakness and uncontrolled and rapid degradation by enhancing the protein network through advanced chemical cross-linking [30,31], or by developing complex hybrid systems [32,33,34]. These modifications often introduce synthetic components or processing steps that may introduce concerns regarding cytotoxicity, inflammatory responses to materials, and a loss of native biological properties. Furthermore, such approaches frequently result in more complex, time-consuming, and costly synthesis processes, limiting their scalability and accessibility in standard clinical or research settings. Another critical factor is the inherent batch-to-batch variability of fibrinogen and thrombin, which often stem from different sourcing or preparation techniques. This inconsistency can significantly affect the reproducibility of hydrogel mechanical and structural properties, posing a challenge for standardized applications [23].

In contrast, this work explores the potential of pure fibrin gels as versatile biomaterials for a wide range of biomedical applications and emphasizes the exceptional biocompatibility and intrinsic physicochemical properties of fibrin gel, which can be fine-tuned by varying the concentrations of fibrinogen and thrombin. Indeed, it is well established that fine-tuning parameters such as precursor concentrations, as well as ionic strength, allows for precise control over the resulting gel morphology, physicochemical properties, and long-term stability [35,36,37]. Specifically, this work aims to characterize the mechanical and morphological characteristics of fibrin gels, examine the relationship between reagent concentrations and structural integrity, and assess their impact on swelling behavior, water absorption, and stability. Additionally, this research aims to optimize the synthesis protocol for pure fibrin-based hydrogels, validating it through comprehensive physical and chemical characterization. Ultimately, the goal is to propose these materials as adaptable solutions for specific biomedical needs.

## 2. Results and Discussion

As a biomaterial, fibrin shows substantial advantages as a hydrogel compared to other natural or synthetic alternatives. Since fibrin gel is directly linked with the blood-clotting cascade, it intrinsically addresses many requirements of biomaterials from a biological perspective. It exhibits noteworthy biological relevance, biocompatibility, and biodegradability, as well as the ability to preserve encapsulated cells and bioactive molecules during hydrogel production. Moreover, fibrin hydrogels present tunable mechanical and structural properties; the stiffness, porosity, and fiber architecture of the fibrin network can be finely adjusted by modifying the concentrations of fibrinogen and thrombin during gelation [24,25,26,27]. This adaptability is central to our study, which systematically explores how variations in fibrinogen and thrombin concentrations affect the physicochemical, mechanical, and cytocompatibility properties of these hydrogels [35,36,37]. Particularly, the concentrations utilized in this study are summarized in Table 1, reported in Section 4.1. The concentration ranges were chosen to remain within the physiological range: fibrinogen concentration above 1 mg/mL allows for blood clot formation in the blood vessels [38]; the physiological concentration of fibrinogen in plasma is between 1.2 and 4 mg/mL [39,40]; thrombin physiological concentration ranges from 0.1 to 10 U/mL [41]; and Ca^2+^ physiological concentration ranges between 1.17 and 1.40 mM [42].

### 2.1. Infrared (IR) Spectroscopy

**Figure 1:**
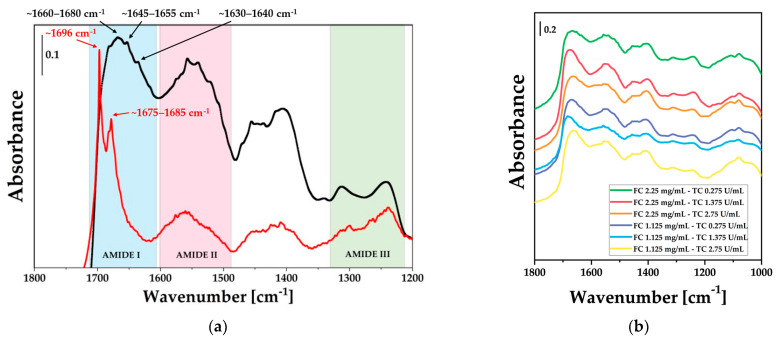
(**a**) DRIFT spectra comparing the bovine fibrinogen powder (black line) and a representative fibrin gel formulation (FC2.25-TC1.375) (red line) and (**b**) DRIFT spectra of fibrin gels prepared at different concentrations of fibrinogen working solution (WS) and thrombin WS. The labels indicate the final concentrations of fibrinogen (FC, mg/mL) and thrombin (TC, U/mL) achieved after mixing the respective WS components.

Firstly, the successful synthesis of fibrin gel was confirmed through the comparison of IR spectra of fibrinogen and fibrin gel. As shown in Figure 1a, evident changes in the spectral features were observed when comparing the spectrum of the precursor fibrinogen to that of the fibrin gel obtained after thrombin-catalyzed polymerization. In particular, in the amide I region between 1700 and 1600 cm^−1^, broadening of the signals was observed as the fibrin gel forms, due to a loss of homogeneity. This behavior reflects a structural rearrangement as fibrinogen converts to a fibrin network, as already mentioned by Dutta et al. [43]. Moreover, a more detailed analysis of the amide I region provides insights into the secondary structure transitions during the fibrinogen-to-fibrin conversion. Fibrinogen spectrum is characterized by high-frequency peaks (~1700–1675 cm^−1^) associated with β-turns and anti-parallel β-structures [44]. Upon thrombin-catalyzed polymerization, a significant structural rearrangement occurs, as evidenced by the emergence of peaks at approximately 1650 cm^−1^ and 1640–1630 cm^−1^. The signal at ~1650 cm^−1^ is the signature of the α-helical domains preserved from the native coiled-coil structure, while the peak at ~1635 cm^−1^ serves as a marker for the formation of inter-chain β-sheets [44]. These spectral variations confirm the stabilization of the insoluble fibrin network due to the formation of intermolecular hydrogen bonds. Figure 1b compares the IR spectra of the different fibrin gels in the fingerprint spectral region, which shows the characteristic amide I band at ∼1650 cm^−1^, amide II peak at ∼1550 cm^−1^, and amide III signal at ∼1250 cm^−1^ [45]. Focusing on the amide I band, which was particularly sensitive to hydrogen bonding, it can be observed that its peak position shifted to 1680 and 1660 cm^−1^ depending on the specific sample composition.

### 2.2. Scanning Electron Microscopy (SEM) Results

**Figure 2:**
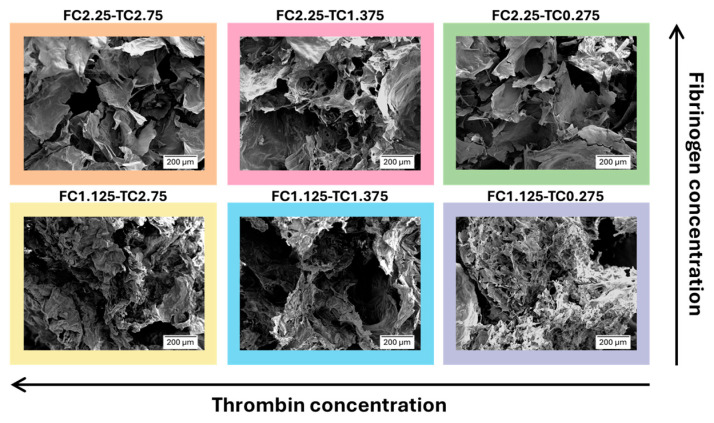
SEM images of fibrin gel obtained with different concentrations of fibrinogen WS and thrombin WS.

Scanning Electron Microscopy (SEM) images of the internal microstructures of freeze-dried fibrin gels with varying concentrations of fibrinogen and thrombin are presented in Figure 2.

Both fibrinogen WS and thrombin WS concentrations distinctly modify the internal structure of fibrin gels. Particularly, increasing fibrinogen WS and thrombin WS concentrations resulted in an internal microstructure characterized by fewer fibers, less interconnection, and larger pores. The observation that high fibrinogen concentrations result in larger and interconnected pores may seem contradictory; however, similar findings have been reported by Zhao et al. [46]. A possible explanation lies in the competitive rates of fibrinopeptide cleavage and lateral aggregation already discussed by Ryan et al. [47]. In environments with high fibrinogen and/or thrombin availability, the fibrin system can undergo rapid and non-equilibrium aggregation, resulting in the formation of isolated fibers and larger interstitial voids. Furthermore, according to Domingues et al. [48], high thrombin concentrations significantly impair protofibril packing. This reduced internal density may prevent fibers from reaching the structural integrity required to form stable and spread interconnections, forcing the network into a metastable state where the fibrinogen fibers are distributed unevenly.

### 2.3. Rheological Analysis

**Figure 3:**
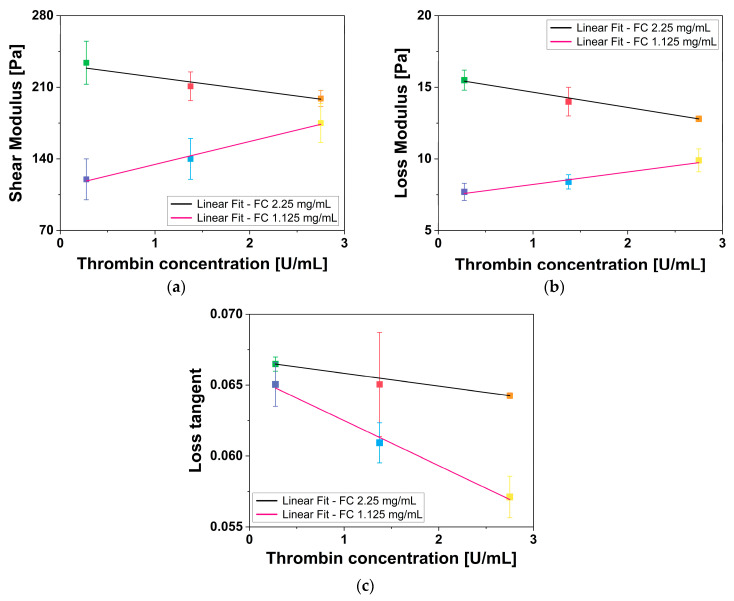
Linear regression fits of (**a**) shear modulus, (**b**) loss modulus, and (**c**) loss tangent of all fibrin gels, having the same fibrinogen WS concentration, as a function of thrombin WS concentration. Error bars represent U.

Rheological measurements were used to extract the shear modulus (G′), the loss modulus (G″) and the loss tangent (tan δ) of fibrin gels with varying concentrations of fibrinogen WS and thrombin WS. G′, G″, and tan δ of all fibrin gels were plotted as a function of thrombin WS concentration, analyzed through a linear regression fit of fibrin gels having the same fibrinogen WS concentration and different thrombin WS concentrations, and the results are reported in Figure 3a, Figure 3b, and Figure 3c, respectively. The measured values of G′, G″, and tan δ align with previous findings by Liu et al. [49], Moreno-Arotzena et al. [50], and Ryan et al. [47].

The rheological characterization of the fibrin gels revealed that the mechanical properties of the network are governed by a complex interplay between fibrinogen density and thrombin-induced polymerization kinetics. Analysis of both G′ and G″ values in relation to thrombin WS concentration through linear fit revealed distinct relationships for the two different fibrinogen WS concentrations. For 2.25 mg/mL fibrinogen WS, both moduli exhibited an inverse proportionality to thrombin WS concentration, with a coefficient of determination (R^2^) and Pearson’s correlation coefficient (PCC) equal to R^2^ = 0.879 and PCC = −0.969 for G′ and R^2^ = 0.991 and PCC = 0.997 for G″. The inverse relationship observed in the 2.25 mg/mL samples aligns with the mechanism proposed by Domingues et al. [48] in whereby high thrombin levels induce a weakening of the gel structure. In a fibrinogen high-density environment, a rapid polymerization induced by high thrombin levels can result in a kinetically trapped metastable state characterized by reduced interconnections among fibers. This phenomenon was explicitly confirmed by our SEM analysis, which revealed a lower branchpoint density in samples prepared with the higher thrombin concentrations. This reduction in network connectivity directly accounts for the observed decline in G′ as a less interconnected scaffold diminishes its energy storage capability, leading to the observed decline in stiffness.

Contrary, 1.125 mg/mL fibrinogen WS exhibited a direct proportionality for both moduli (G′: R^2^ = 0.979 and PCC = 0.995 and G″: R^2^ = 0.935 and PCC = 0.983). In this context, thrombin appears to act as a limiting factor for basic network connectivity, reinforcing a scaffold that would otherwise remain mechanically unstable. This supports a biphasic response model in which the role of thrombin shifts from a structural stabilizer in dilute systems to a factor inducing structural inefficiency in higher-density systems, as already mentioned by Domingues et al. [48] and Ryan et al. [47]. Moreover, lower fibrinogen WS concentrations consistently resulted in lower values of G′, indicating a reduction in the overall mechanical stiffness of the network.

Across all tested conditions, G′ remained consistently higher than G″ with tan δ values consistently below 0.1. It suggests that all the tested materials have a predominantly elastic behavior with a minor viscous component, typical of well-structured hydrogels [51]. Despite the opposite trends observed at different fibrinogen WS concentrations, the tan δ exhibited an inverse proportionality to thrombin content across all tested fibrinogen concentrations (FC 2.25 mg/mL: R^2^ = 0.998 and PCC = −0.999 and FC 1.125 mg/mL: R^2^ = 0.984 and PCC = −0.996). The generalized slight decrease in tan δ suggests a shift toward a more elastically dominant behavior as thrombin concentration increases, suggesting that the network becomes more energetically efficient as thrombin concentration rises. In agreement with the internal dynamics described by Domingues et al. [48], the protofibril arrangement could be the primary regulator of network viscoelasticity. It is probable that higher thrombin levels facilitate an internal fiber architecture characterized by reduced viscous friction or increased resistance to protofibril sliding. Furthermore, a more dilute scaffold may favor a more uniform stress distribution across fewer fibers. This results in a structurally optimized response that may reduce relative viscous dissipation compared to the higher-density counterparts. This suggests that thrombin may influence the effectiveness of the elastic response independently of network connectivity and overall mechanical stiffness.

### 2.4. Swelling Capabilities

**Figure 4:**
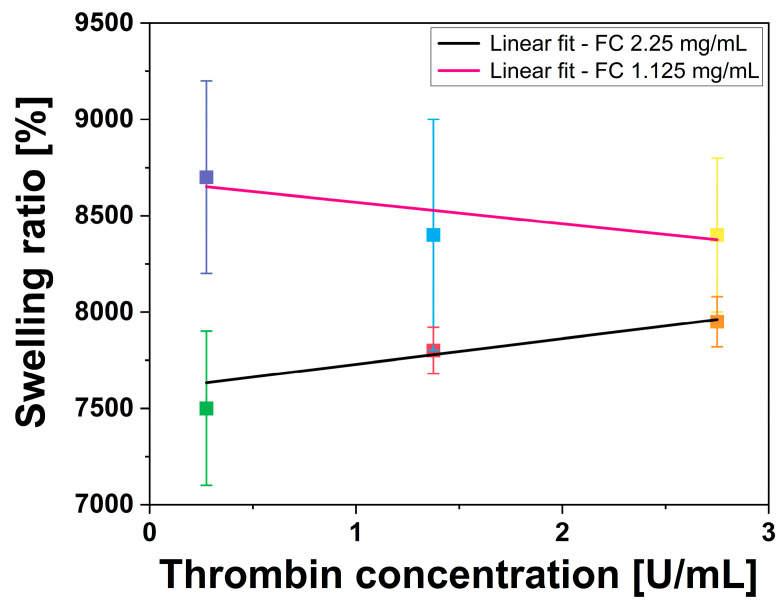
Linear fit of swelling ratio of all fibrin gels, having the same fibrinogen WS concentration, as a function of thrombin WS concentration. Error bars represent U.

The effect of fibrinogen WS and thrombin WS concentrations on swelling capabilities of fibrin gels was estimated by plotting the swelling ratio percentage as a function of thrombin WS concentration and performing a linear regression analysis of fibrin gels prepared with the same fibrinogen WS concentration, but different thrombin WS concentrations (Figure 4). The obtained swelling ratios, ranging from approximately 7000% to 9000%, fall within the numerical range reported by Tan et al. [52] (6830–9770%) for fibrin gels, by Gao et al. [53] for other natural hydrogels, and Dohi et al. [54] for synthetic hydrogels. This confirms that the fibrin hydrogels produced in this study maintain a hydration capacity consistent with established fibrin-based benchmarks.

Fibrin gels prepared with 1.125 mg/mL of fibrinogen WS had higher swelling capability compared to those prepared with 2.25 mg/mL of fibrinogen WS. Moreover, the fibrin gels prepared with 2.25 mg/mL fibrinogen WS showed a negative correlation with increasing thrombin concentration, whereas the opposite trend was observed for fibrin gels prepared with 1.125 mg/mL of fibrinogen WS (2.25 mg/mL fibrinogen WS: R^2^ = 0.805, PCC = −0.950, and *p*-value < 0.05; 1.125 mg/mL fibrinogen WS: R^2^ = 0.525, PCC = −0.873, and *p*-value < 0.05).

### 2.5. Water Uptake Capabilities

**Figure 5:**
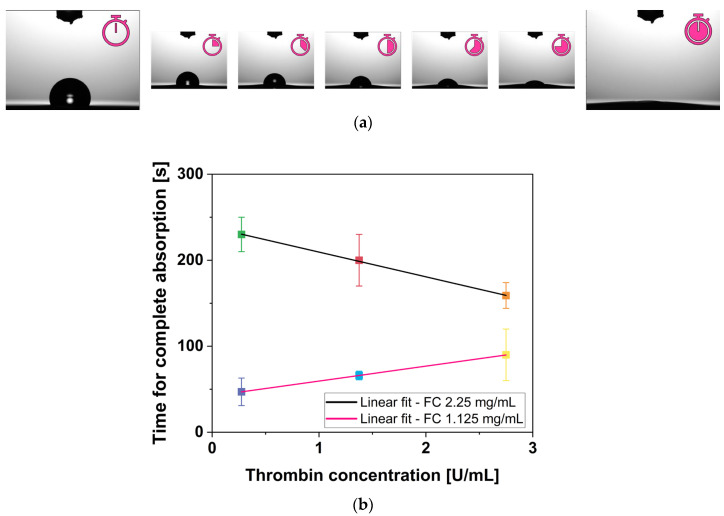
Water uptake capabilities result. (**a**) Qualitative results of a H_2_O drop absorption over time on fibrin gel surface and (**b**) linear fit of H_2_O drop absorption time of all fibrin gels, having the same fibrinogen WS concentration, as a function of thrombin WS concentration. Error bars represent U.

When an H_2_O drop was placed on the surface of a fibrin gel, it was completely absorbed as a function of time, as qualitatively shown in Figure 5a. The time required for complete absorption is dependent on both fibrinogen WS and thrombin WS concentrations. Figure 5b shows the time necessary for fibrin gel to completely absorb the H_2_O drop as a function of thrombin WS concentration. Specifically, fibrin gels with 2.25 mg/mL fibrinogen WS required more time to completely absorb the H_2_O drop with respect to 1.125 mg/mL fibrinogen WS. Moreover, the linear fit of H_2_O absorption as a function of thrombin WS concentration revealed an existing inverse relationship for 2.25 mg/mL fibrinogen WS, whereas a direct relationship was observed for those prepared with 1.125 mg/mL fibrinogen WS. Notably, R^2^ and PCC are both equal to |0.999| for both concentrations of fibrinogen WS, indicating a strong correlation in the observed trends.

These results raise important considerations. Intuitively, one might expect that a less tangled fiber network would facilitate greater swelling and water uptake due to increased deformability linked with reduced mechanical integrity. This paradox highlights the complex interplay between fibrinogen concentration and gel properties. One possible explanation, that remains speculative, is that the lower surface energy associated with higher fibrinogen concentration, observed by Hernández et al. [55], could make the H_2_O uptake process less thermodynamically favorable, increasing the time required for the H_2_O drop to be absorbed. A reasonable consequence is that the fibrin networks with higher fibrinogen content may reach the equilibrium at a lower hydration state, resulting in a reduced swelling ratio, as already noted by Gandhi et al. [56]. Additional experimental investigation would be required to directly confirm this hypothesis.

### 2.6. Gelation Time

**Figure 6:**
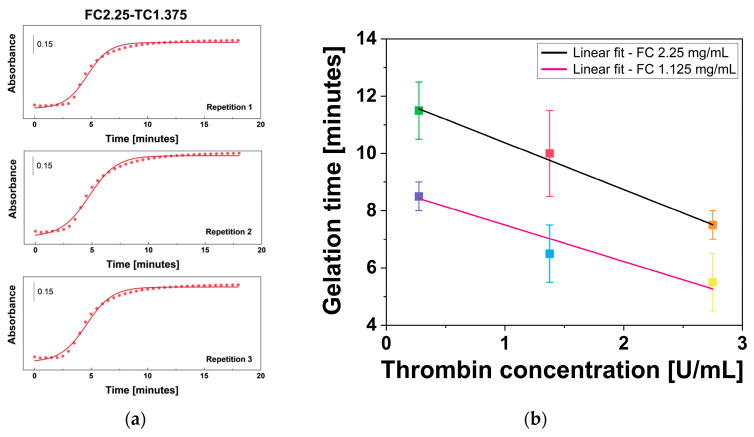
Gelation time results (**a**) sigmoidal fit of absorbance vs. time of FC2.25-TC1.375 sample obtained via UV-Vis spectroscopy, and (**b**) linear fit of fibrin gel gelation time vs. thrombin WS concentration at the same fibrinogen WS concentrations. Error bars represent U.

The role of thrombin in fibrin gel formation is well-established [57]. Thrombin accelerates the conversion of fibrinogen to fibrin, increasing the rate of gelation without becoming consumed in the reaction. In this study, gelation time (*t_gel_*) ranged from 5 to 12 min across the different tested formulations, values that are in close agreement with those reported by Montero et al. [39]. The effect of fibrinogen WS and thrombin WS concentration on *t_gel_* of fibrin gel was estimated by plotting absorbance as a function of time and fitting the data with a sigmoidal regression model. The obtained curve is shown in Figure 6a for sample FC2.25-TC1.375 and in Supplementary file S1 for the other samples (Figures S1–S5). The UV-Vis gelation curve exhibits a characteristic sigmoidal profile, typical of nucleation-dependent polymerization [58]. The initial lag phase corresponds to the enzymatic cleavage of fibrinogen into monomers, followed by a rapid growth phase as protofibrils undergo lateral aggregation into thicker fibers. Finally, the plateau phase indicates the completion of the 3D fibrin network [58]. Subsequently, *t_gel_* of all fibrin gels was plotted as a function of thrombin WS concentration and analyzed through a linear regression fit (Figure 6b).

Analysis of the *t_gel_* values in relation to thrombin WS concentration at the same fibrinogen WS concentrations using linear fit is performed. The relationship between *t_gel_* and thrombin WS concentration indicated that these two parameters are inversely proportional for both fibrinogen WS concentrations (2.25 mg/mL fibrinogen WS: R^2^ = 0.996, PCC = −0.999, and *p*-value < 0.05; 1.125 mg/mL fibrinogen WS: R^2^ = 0.921, PCC = −0.980, and *p*-value < 0.05). This trend underscores the dose-dependent catalytic efficiency of thrombin: higher concentrations accelerate the cleavage of fibrinopeptides A and B, thereby promoting a more rapid transition from fibrinogen monomers to a structured polymer network.

The results support the conclusion that thrombin is the primary kinetic regulator in fibrin gel systems [18,59,60]. Indeed, a 10% increase in thrombin concentration resulted in a notable decrease in *t_gel_* by approximately 35 ± 4% for 2.25 mg/mL fibrinogen and 35 ± 7% for 1.125 mg/mL fibrinogen, suggesting that thrombin enhances the efficiency of gel formation under the tested conditions.

### 2.7. Stability and Degradation over Time

The analysis of the stability over time (SoT) and degradation over time (DoT) was performed to identify the baseline stability of fibrin networks. A common challenge with fibrin-based hydrogel is spontaneous and unpredictable degradation even in the absence of fibrinolytic enzymes. Therefore, demonstrating long-term integrity of both the sol–gel system (hydrated condition) and only-gel content (freeze-dried condition) is fundamental to identifying the formulation that guarantees stability in terms of the sol–gel system and only-gel content of fibrin gel.

**Figure 7:**
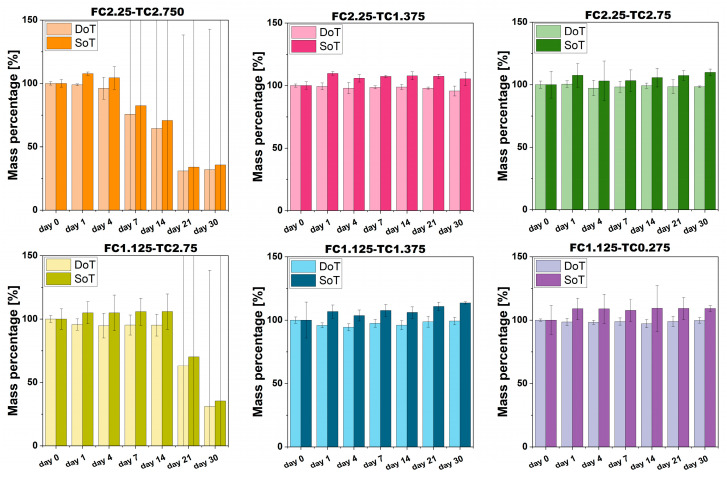
Results of stability over time and degradation over time for fibrin gels obtained at different fibrinogen WS and thrombin WS concentrations. Error bars represent U.

The results of the fibrin gel SoT and DoT are presented in Figure 7. The graph clearly indicates that the fibrin gel maintains its stability under hydrated conditions throughout the 30-day study period within the following ranges: 2.25 mg/mL ≤ FC ≤ 1.125 mg/mL and 1.375 U/mL ≤ TC ≤ 0.275 U/mL. Notably, the fibrin gel did not show any signs of degradation when prepared with fibrinogen WS and thrombin WS concentrations within the same ranges.

Moreover, it is important to note that the SoT and DoT of the fibrin gels are significantly influenced by the concentration of thrombin WS. Specifically, thrombin concentrations exceeding 1.375 U/mL resulted in instability of the fibrin gel, while all other formulations tested remained stable for at least 30 days. This macroscopic failure can be directly attributed to the kinetically trapped metastable state [47,48] previously discussed. An excessively rapid polymerization rate, driven by high thrombin WS concentration, can prevent effective fibrin fiber organization. Among the tested concentrations, higher thrombin levels could induce a network failure, as the system remains in a metastable state that is inherently susceptible to spontaneous collapse and premature degradation. These findings suggest that the enzymatic rate may be more than a factor for modulating fibrin gel properties, potentially serving as a key influence on long-term structural integrity. Achieving a balanced thrombin-to-fibrinogen ratio appears to be beneficial in avoiding metastable configurations, thereby supporting the development of a more cohesive and durable scaffold for in vitro applications.

### 2.8. Cytocompatibility Evaluation

**Figure 8:**
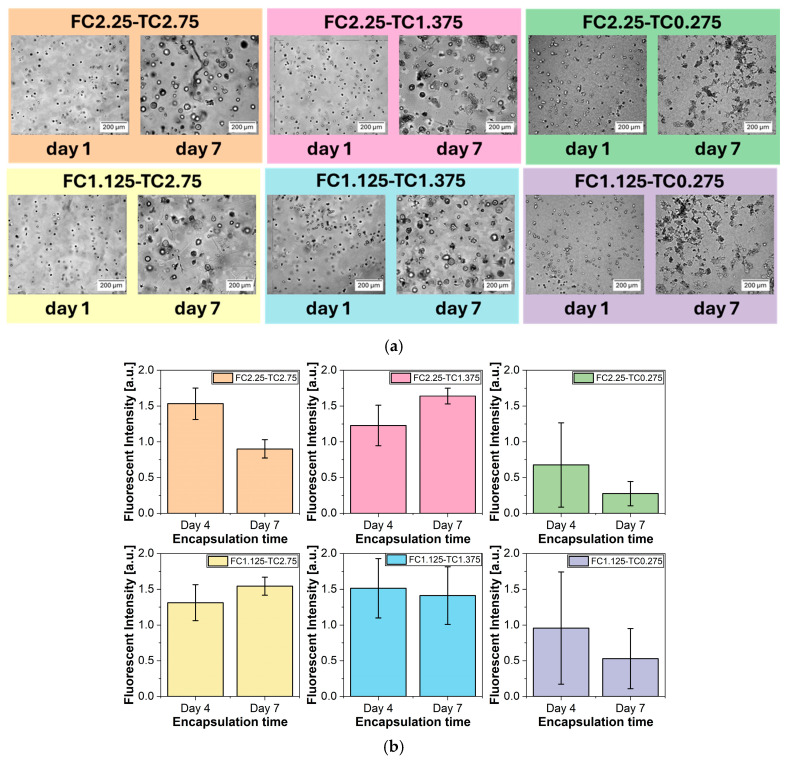
Results of (**a**) qualitative assessment of cytocompatibility. Optical images of each fibrin gel sample with A549 cells embedded in it at day 1 and day 7 of incubation, and (**b**) resazurin-based viability assay of A549 cells embedded in each fibrin gel sample. Each graph represents fluorescence intensity at day 4 and day 7 of A549 cells incubation in fibrin gels. Error bars represent U.

Optical microscopy images, acquired to qualitatively assess the cytocompatibility of fibrin gels, are presented in Figure 8a. A549 cells embedded in fibrin gels with a thrombin WS concentration of 0.275 U/mL exhibited marked morphological deterioration after 7 days, indicating cytotoxic effects of the material. In contrast, fibrin gels with higher thrombin WS concentrations (1.375 U/mL and 2.75 U/mL) did not induce morphological alterations in A549 cells. Instead, A549 cells were able to grow in compact spheroids, indicating both viability and proliferating activity when embedded in fibrin gel matrices.

Resazurin-based viability assay results are illustrated in Figure 8b. These findings corroborated the qualitative observations, revealing a decrease in the metabolic activity and number of A549 cells embedded in fibrin gel with 0.275 U/mL thrombin WS, thereby confirming the cytotoxic effects of this formulation. At 0.275 U/mL thrombin WS concentration, slower polymerization kinetics appear to favor a more densely interconnected network, characterized by thinner fibers and reduced pore sizes. While structurally stable, this configuration could potentially restrict the efficient diffusion of nutrients and the removal of metabolic byproducts. The high fiber density and reduced pore diameter may impose steric hindrance, potentially trap larger macromolecular components and limit their effective transport through the scaffold. This hypothesized limited mass transport might result in a localized microenvironment unfavorable for long-term cell survival, contributing to the observed decline in metabolic activity over the 7-day period. In contrast, all other tested concentrations of thrombin WS, leading to larger pores, demonstrated cytocompatibility with A549 cells, supporting their suitability for cell-embedded fibrin gel systems.

The ability to modify the overall properties of fibrin gels by adjusting the concentrations of fibrinogen and thrombin positions these gels as promising and versatile candidates for various biomedical applications. Our work specifically aims to identify the optimal combination of fibrinogen and thrombin that ensures the best balance of physicochemical properties and cytocompatibility within an in vitro experimental framework, making this material a potential candidate for several biomedical applications. Within the range of concentrations investigated in this work, the FC2.25-TC1.375 formulation was found to offer the most favorable balance of physical, chemical, and biological properties. Its composition not only promotes robust cell viability but also delivers the mechanical stability and favorable physicochemical properties essential for a variety of therapeutic uses. These findings confirm the adaptability of fibrin-based hydrogels for applications such as wound healing [61], tissue regeneration [62,63], localized drug delivery [64], and the development of 3D in vitro models [65]. This adaptability reinforces the potential of the FC2.25-TC1.375 formulation in advanced biomedical technologies, positioning it as a promising option for several therapeutic strategies [16,66,67].

However, this study is limited to static in vitro conditions and a specific range of fibrinogen and thrombin concentrations. Future studies should investigate functional cellular responses and performance under dynamic or in vivo-like conditions to further validate these findings.

## 3. Conclusions

This study highlighted the crucial role of fibrinogen and thrombin in tailoring the performance of fibrin gels, demonstrating their significance as adaptable biomaterials for several biomedical applications. Our systematic investigation reveals that the formulation comprising 2.25 mg/mL fibrinogen and 1.375 U/mL thrombin (FC2.25-TC1.375) provides a highly favorable balance between mechanical stability, architectural homogeneity, and cytocompatibility. Within the experimental range tested, this specific ratio appears optimal for maintaining structural integrity while preserving the high bioactivity required for A549 cell viability.

Moving beyond simple characterization, these findings highlight that stability and reproducibility in pure fibrin systems can be achieved without the need for complex chemical modifications. This suggests that pure fibrin, when precisely formulated, remains a powerful and accessible tool for advanced biomedical technologies, such as wound healing, localized drug delivery, and the development of 3D in vitro models.

Future research should focus on evaluating the long-term remodeling of this optimized fibrin gel formulation under dynamic physiological conditions and its performance when integrated with specific cell lines or growth factor release profiles. While this study establishes a robust baseline for reproducible fibrin-based scaffolds, further exploration into the degradation kinetics in vivo will be essential to fully translate these adaptable hydrogels into standardized clinical therapies.

## 4. Materials and Methods

### 4.1. Fibrin Gel Synthesis

The synthesis of fibrin gel was carried out according to a protocol previously developed and optimized by our research group [68], fully described in Supplementary file S2. Bovine fibrinogen (Fraction I, type I-S from bovine plasma, Sigma-Aldrich, St. Louis, MO, USA) was dissolved in a 0.9% sodium chloride (NaCl) solution (CARLO ERBA Reagents, Cornaredo, Italy) at 37 °C to achieve concentrations of either 5 mg/mL or 2.5 mg/mL (Fibrinogen WS). The use of 0.9% NaCl solution as a solvent was specifically intended to maintain a constant physiological ionic strength during the initial protein dissolution. Thrombin powder derived from bovine plasma (Sigma-Aldrich, St. Louis, MO, USA) was reconstituted in MilliQ-H_2_O to obtain a final concentration of 100 U/mL. Following complete dissolution, each solution was sterilized using a 0.22 µm filter. A 50 mM calcium chloride (CaCl_2_) solution (Sigma-Aldrich, St. Louis, MO, USA) was prepared in MilliQ-H_2_O and sterilized via autoclaving.

The thrombin solution (100 U/mL), CaCl_2_ solution, and MilliQ-H_2_O were mixed in the following ratios: 1:1:0, 1:2:1, and 1:10:9, resulting in intermediate thrombin concentrations of 50 U/mL, 25 U/mL, and 5 U/mL, respectively, along with a CaCl_2_ concentration of 25 mM. To dilute the thrombin-CaCl_2_ solution by one-tenth, DMEM culture medium supplemented with 10% heat-inactivated fetal bovine serum (FBS) (Sigma-Aldrich, St. Louis, MO, USA), 2 mM glutamine (Lonza, Basel, Switzerland), and 1% penicillin/streptomycin (Sigma-Aldrich, St. Louis, MO, USA) was added (referred to as thrombin WS). The choice of a cell culture medium with 10% FBS as a diluent was strategically made to ensure an immediate nutrient-rich environment for cells during the encapsulation process and to provide a robust bicarbonate buffering system, ensuring that the pH of the final hydrogel was maintained at a physiological value of around 7.4.

Fibrin gel was formed through a sol–gel reaction by mixing Fibrinogen WS and Thrombin WS in a 9:11 ratio, resulting in fibrinogen concentrations of 2.25 mg/mL or 1.125 mg/mL, thrombin concentrations of 2.75 U/mL, 1.375 U/mL, and 0.275 U/mL, and a CaCl_2_ concentration of 1.375 mM (Table 1). To ensure stable pH and temperature consistency during polymerization, the samples were immediately transferred to a humidified incubator at 37 °C with a 5% CO_2_ atmosphere. This controlled environment allowed the bicarbonate in the medium to stabilize the pH at 7.4 and guaranteed reproducible gelation conditions across all experimental groups.

**Table 1:** Different concentrations of precursors—fibrinogen, thrombin, and CaCl_2_—tested to synthesize fibrin gel.

Sample Name	Fibrinogen Concentration [mg/mL]	Thrombin Concentration [U/mL]	CaCl_2_ Concentration [mM]
FC2.25-TC2.75	2.25	2.75	1.375
FC2.25-TC1.375	2.25	1.375	1.375
FC2.25-TC0.275	2.25	0.275	1.375
FC1.125-TC2.75	1.125	2.75	1.375
FC1.125-TC1.375	1.125	1.375	1.375
FC1.125-TC0.275	1.125	0.275	1.375

### 4.2. Infrared (IR) Spectroscopy

1 mL of fibrin gel was synthesized in a 1.5 mL microcentrifuge tube. Following cross-linking, the hydrogels were equilibrated in PBS overnight at 37 °C to attain their equilibrium water content. Subsequently, samples were subjected to three 5-min washes in dH_2_O (distilled water), then frozen at −20 °C and lyophilized (freeze-dried). Freeze-dried samples, with an average weight of 5 mg, were analyzed using a Bruker Equinox 55 spectrometer (Bruker, Billerica, MA, USA) equipped with a mercury cadmium telluride detector and a Spectra Tech DRIFT accessory (model 0030-011) (Spectra Tech, Chakan, MA, USA). IR spectra were acquired in diffuse reflectance (DRIFT) mode from 4000 to 600 cm^−1^. Spectra were collected under dry air conditions by averaging 64 interferograms at a spectral resolution of 4 cm^−1^. The recorded reflectance data were subsequently transformed into pseudo-absorbance values: A = −log(R), where R represents the measured reflectance.

### 4.3. Scanning Electron Microscopy (SEM)

The internal microstructure of fibrin gels was investigated through SEM. 1 mL of fibrin gel was synthesized in a 1.5 mL microcentrifuge tube. Following cross-linking, the hydrogels were equilibrated in PBS overnight at 37 °C to attain their equilibrium water content. Subsequently, samples were subjected to three 5-min washes in dH_2_O, then frozen at −20 °C and lyophilized (freeze-dried). Freeze-dried specimens were rapidly immersed in liquid nitrogen, fractured, and sputter-coated with gold using AGB7234 high-resolution sputter (Agar Scientific, Sheffield, UK). SEM imaging of fracture surfaces was performed using a TESCAN VEGA (TESCAN Orsay Holdings, Brno, Czech Republic) SEM platform operated at 3 kV.

### 4.4. Rheological Analysis

The rheological characterization of fibrin gel was carried out using a Thermo Scientific Rheostress 6000 rheometer (Thermo Fisher Scientific, Waltham, MA, USA), employing the oscillatory test method, and measuring the shear strain. 4 mL of fibrin gel was synthesized, allowed to crosslink overnight, and tested in the lid of a Petri dish with a 30 mm diameter. The geometry used was the serrated plate–plate (P35 Ti L) with a diameter of 35 mm (PP35). The gap between the plates was adjusted based on the applied normal force (0.1 N). All measurements were taken at temperatures between 23 °C and 25 °C by setting and maintaining 1 Hz for frequency and 0.5 Pa of shear stress. All conditions were tested in triplicate (*n* = 3), and results are reported as mean ± U, where U was the expanded uncertainty.

### 4.5. Gelation Time

The gelation kinetics of fibrin gels were quantitatively assessed using UV-Vis spectroscopy with a Spark multimode microplate reader (Tecan Trading AG, Männedorf, Switzerland). The effects of thrombin and fibrinogen concentrations on the gelation process were evaluated by monitoring absorbance at 350 nm, with readings taken every 30 s over a total duration of 2 h at 37 °C. Specifically, 90 µL of fibrinogen WS was added to a 96-well plate, and 110 µL of thrombin WS was introduced immediately prior to measurements in the pre-warmed spectrophotometer. All conditions were tested in triplicate (*n* = 3), and results are reported as mean ± U.

The absorbance versus time curve had an S-shape, and the gelation kinetics were described by a four-parameter sigmoidal function, which models a smooth, monotonic transition from an initial state to a final state and is characterized by an inflection point and a steepness parameter. The *t_gel_*, defined as the moment when the gelation process is deemed complete, was identified as the point at which 99% of the maximum height of the sigmoidal absorbance curve was reached. It was preferred to the inflection point that represents the characteristic time of the rapid assembly phase but not to the practical endpoint of gel maturation. To determine *t_gel_*, the sigmoidal curve obtained from UV-Vis data was fitted using Equation (1).

(1) $$y=\frac{A_{1}+A_{2}}{1+e^{\frac{x-x_{0}}{dx}}}+A_{2}$$

where *A*_1_ represents the upper asymptote, *A*_2_ the lower asymptote, *x*_0_ is the inflection point, and dx is the steepness of the curve. After the estimation of the parameters *A*_1_, *A*_2_, *x*_0_, and *dx*, *t_gel_* was calculated using Equation (2).

(2) $$t_{gel}=x_{0}-\left[dx\cdot \ln\left(\frac{0.01\cdot A_{1}-0.01\cdot A_{2}}{0.99\cdot A_{1}-0.99\cdot A_{2}}\right)\right]$$

### 4.6. Swelling Ratio

Swelling capabilities of fibrin gel were tested by evaluating the sol–gel fraction, which refers to the ratio of the sol (liquid content) to the gel (a cross-linked, network-like polymer) in a material undergoing the sol–gel transition. 1 mL of fibrin gel was synthesized inside 1.5mL microcentrifuge tube; the net mass of fibrin gel was determined by subtracting the tare mass (test tube) from the gross mass (test tube + fibrin gel). The net mass of fibrin gel under hydrated conditions (*m_hydrated_*) was collected. All the samples were subjected to three 5-min washes in dH_2_O, then frozen at −20 °C and freeze-dried. The net mass of fibrin gel in dehydrated condition (*m_dehydrated_*) was calculated as done for hydrated samples. The swelling ratio (SR) percentage was calculated using Equation (3).

(3) $$SR\;[\%]=\frac{m_{hydrated}-m_{dehydrated}}{m_{dehydrated}}\cdot 100$$

All conditions were tested in triplicate (*n* = 3), and results are reported as mean ± U.

### 4.7. Water Uptake Capabilities

The water uptake capabilities of fibrin gels were evaluated using an optical tensiometer (Attension^®^ Theta Lite, Biolin Scientific, Gothenburg, Sweden). 1 mL of fibrin gel was uniformly spread onto glass slides to create a smooth, flat surface for testing. A drop of dH_2_O was then placed on the surface of the samples. The interaction between the water drop and the fibrin gel was monitored by recording a video over 120 s at a rate of 10 frames per second, and a Python-based image analysis script (version 3.12.9) was developed and utilized to analyze the frames. All conditions were tested in triplicate (*n* = 3), and results are reported as mean ± U.

### 4.8. Stability and Degradation Rate over Time

The SoT of fibrin gels was evaluated by measuring mass variations in the fibrin gel under hydrated conditions over a 30-day period. 1 mL of fibrin gel was synthesized in a 1.5 mL microcentrifuge tube, and the net mass of the fibrin gel was determined by subtracting the tare mass (test tube) from the gross mass (test tube plus fibrin gel). Gels were incubated in 250 µL of complete DMEM (supplemented with 10% FBS) at 37 °C and 5% CO_2_ in a humidified incubator. Tubes were kept closed to minimize evaporation, and the medium volume was restored to 250 µL at each time point. The net mass of the fibrin gels was recorded on days 0, 4, 7, 14, 21, and 30. Before weighing, the culture medium was carefully removed, and the inner walls of the tube were gently blotted to remove excess liquid without disturbing the gel. The percentage of mass variation compared to the initial mass over time was calculated using Equation (4).

(4) $$Mass\;variation\;[\%]=\frac{m_{x}}{m_{0}}\cdot 100$$

where m_0_ represents the mass of the sample at day 0, and m_x_ denotes the mass of the sample on the test day (e.g., days 4, 7, 14, 21, and 30). All conditions were tested in triplicate (*n* = 3), and results are reported as mean ± U.

Subsequently, all the samples underwent three 5-min washes in dH_2_O, were then frozen at −20 °C, and freeze-dried. After freeze-drying, the DoT of fibrin gels was assessed by repeating the weight of freeze-dried samples to investigate mass variations in the gel content over time using Equation (4).

### 4.9. Cytocompatibility Analysis

The cytocompatibility of fibrin gels was evaluated by embedding the A549 cells, human lung adenocarcinoma cell line (CCL-185, ATCC), and assessing cell viability over time through both qualitative and quantitative methods. Qualitative assessments were conducted using optical microscopy, while quantitative evaluations were performed using a resazurin-based viability assay.

Fibrin hydrogels were synthesized in a 48-well plate, where two distinct layers of fibrin gel were created in each well. The bottom layer, which did not contain A549 cells, was formed by mixing 90 µL of fibrinogen WS with 110 µL of thrombin WS. This mixture was then incubated at 37 °C with 5% CO_2_ for approximately 30 min to allow for complete gelation. Following the solidification of the bottom layer, a top layer was formed by adding 180 µL of fibrinogen WS and 220 µL of thrombin WS, which contained a cell density of 5·10^4^ cells/cm^3^. This top layer was incubated overnight at 37 °C to facilitate proper embedding of the A549 cells within the fibrin gel matrix.

The following day, 200 µL of complete DMEM (10% FBS) was added to the surface of each fibrin gel scaffold, and this medium was refreshed every 3 to 4 days to maintain optimal cell growth conditions.

Cell viability was assessed at three time points: day 1, day 4, and day 7. Different identical fibrin gel samples were prepared for each testing day, as it was determined that conducting time-lapse experiments with more than two resazurin incubations is not advisable when cells are embedded in fibrin gel [69].

For qualitative analysis, images of the A549 cells embedded in the fibrin gels were captured using a Zeiss Axio Observer Z1/7 inverted microscope (Carl Zeiss, Oberkochen, Germany) equipped with a 10× objective in phase contrast mode. Image analysis was performed using Zeiss software ZEN 2.6 (blue edition) to evaluate cell morphology and distribution within the gel.

For quantitative analysis, the complete DMEM (10% FBS) was removed from the scaffold surfaces, and 200 µL of 44 µM resazurin solution was added to each well for testing. The 48-well plate was incubated at 37 °C with 5% CO_2_ for 3 h to allow for the conversion of resazurin to resorufin by viable cells. Subsequently, 100 µL of the resazurin solution was transferred to a 96-well plate for measurement of fluorescent intensity using a Spark multimode microplate reader (Tecan, Männedorf, Switzerland). The excitation and emission wavelengths were set at 545 nm and 590 nm, respectively. To ensure the reliability of the metabolic activity measurements, a two-step normalization of the fluorescence data was performed. First, the raw values were corrected by subtracting the background signal from cell-free fibrin gel controls (processed in triplicate for each concentration and time point) to account for any potential interference or light scattering from the fibrin scaffold. Subsequently, to evaluate cell proliferation over time, the net fluorescence data obtained on day 4 and day 7 were normalized against the baseline values recorded at day 1. This procedure is fully described and validated by Petiti et al. [69].

### 4.10. Statistical Analysis and Measurement Uncertainty Evaluation

Statistical analyses were performed using Origin 2022 software (OriginLab Corporation, Northampton, MA, USA) and custom Python scripts specifically developed for data analysis. The results are presented as the mean values ± expanded uncertainty (U), providing a comprehensive understanding of the data’s reliability. U was calculated according to the guidelines outlined in the Guide to the expression of Uncertainty in Measurement (GUM) [70,71]. The GUM approach is the international standard for evaluating and quantifying measurement uncertainty, treating both random and systematic errors on a probabilistic basis. It provides a structured and multi-step process to model measurement, identify error sources, and combine them into a comprehensive uncertainty value [72]. In this study, the standard deviation (SD) of repeated measurements was assessed, the combined standard uncertainty (u) was calculated through error propagation theory while considering both SD and, if applicable, instrument uncertainty, and U was obtained by multiplying u by a coverage factor (k) of 2, which corresponds to a confidence level of approximately 95%.

## Data Availability

Data will be available on request.

## Supplementary Materials

The following supporting information can be downloaded at: https://www.mdpi.com/article/10.3390/gels12050351/s1, Supplementary file S1: Results of gelation time; Supplementary file S2: Detailed protocol for fibrin gel synthesis.

## Abbreviations

The following abbreviations are used in this manuscript:

NTE	Natural Tissue Environment
Na^+^	Sodium ions
Ca^2+^	Calcium ions
IR	Infrared
SEM	Scanning Electron Microscopy
G′	Shear modulus
G″	Loss modulus
tan δ	Loss tangent
R^2^	Coefficient of determination
PCC	Pearson’s correlation coefficient
NaCl	Sodium chloride
CaCl_2_	Calcium chloride
FBS	Fetal Bovine Serum
DRIFT	Diffuse reflectance
*t_gel_*	Gelation time
SoT	Stability over Time
DoT	Degradation over Time
GUM	Guide to the expression of Uncertainty in Measurement
U	Expanded uncertainty
SD	Standard Deviation
u	Standard Uncertainty
k	Coverage factor
dH_2_O	Distilled water
