# Supplementary material for: Fibrin Gel as a Versatile Biomaterial Platform in the Biomedical Landscape: Chemical, Physical, and Biological Insights
Source: Gels. 2026 Apr 22;12(5):351. doi: 10.3390/gels12050351 (PMC13205765; doi:10.3390/gels12050351)
Supplement: Supplementary file 1 [file gels-12-00351-s001.zip › Supplementaryfile_S1_gels.pdf]

Supplementary file S1:
Results of gelation time

Results of the gelation process analysis obtained by monitoring absorbance at 350 nm, with readings taken every 30 seconds over a total duration of 2 hours at 37 °C. In Figures S.1-S.5 are reported the sigmoidal fits obtained from UV-Vis data. Sample names refer to fibrin gels prepared with different Fibrinogen WS and Thrombin WS:

FC2.25-TC2.75: fibrinogen concentration 2.25 mg/mL and thrombin concentration 2.75 U/mL.

FC2.25-TC0.275: fibrinogen concentration 2.25 mg/mL and thrombin concentration 0.275 U/mL.

FC1.125-TC2.75: fibrinogen concentration 1.125 mg/mL and thrombin concentration 2.75 U/mL.

FC1.125-TC1.375: fibrinogen concentration 1.125 mg/mL and thrombin concentration 1.375 U/mL.

FC1.125-TC0.275: fibrinogen concentration 1.125 mg/mL and thrombin concentration 0.275 U/mL.

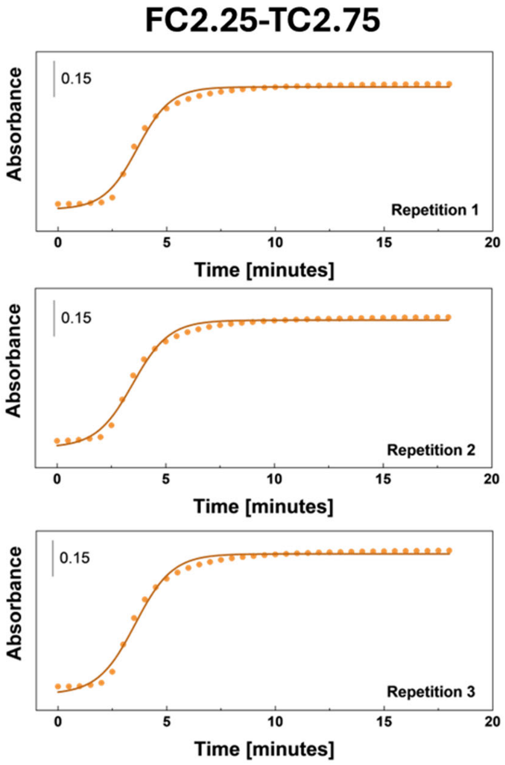

Figure S1. Sigmoidal fit of absorbance vs. time of FC2.25-TC2.75 sample obtained via UV-Vis spectroscopy.

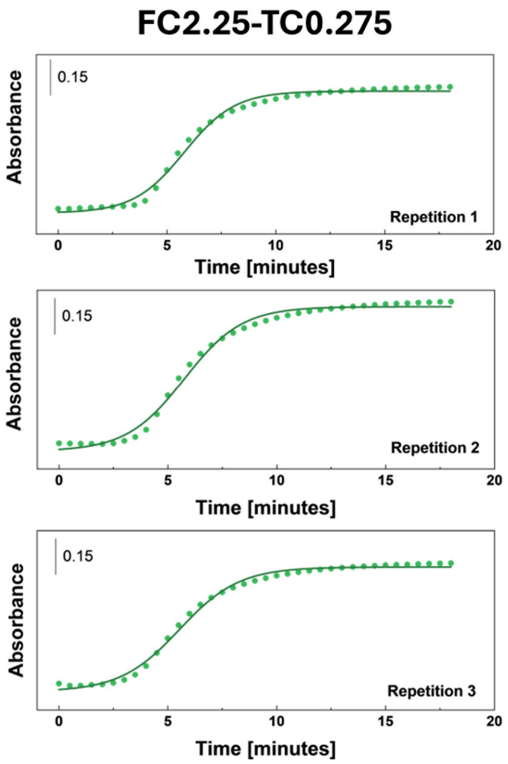

Figure S2. Sigmoidal fit of absorbance vs. time of FC2.25-TC0.275 sample obtained via UV-Vis spectroscopy.

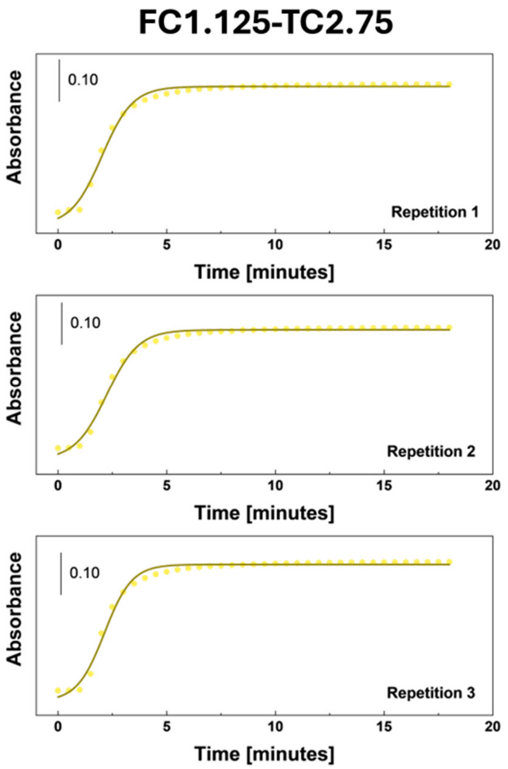

Figure S3. Sigmoidal fit of absorbance vs. time of FC1.125-TC2.75 sample obtained via UV-Vis spectroscopy.

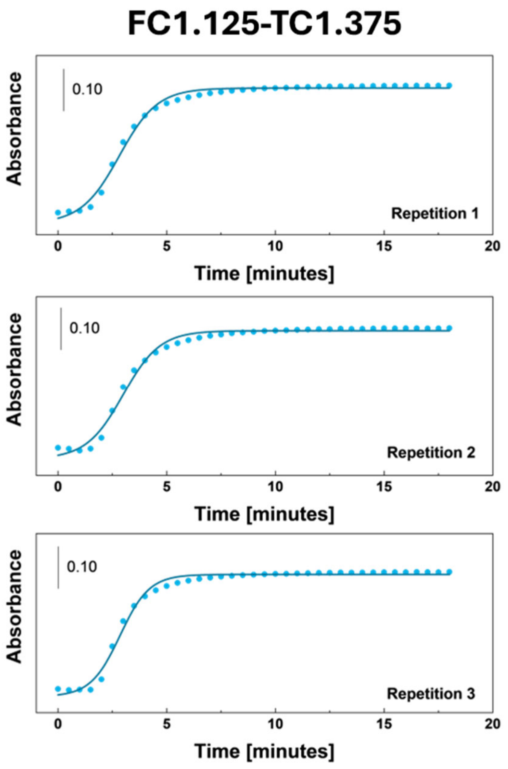

Figure S4. Sigmoidal fit of absorbance vs. time of FC1.125-TC1.375 sample obtained via UV-Vis spectroscopy.

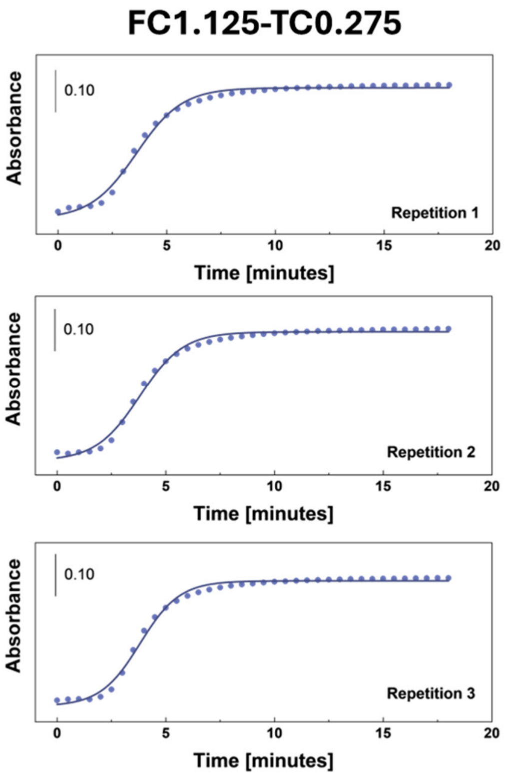

Figure S5. Sigmoidal fit of absorbance vs. time of FC1.125-TC0.275 sample obtained via UV-Vis spectroscopy.
