# Supplementary material for: Fibrin Gel as a Versatile Biomaterial Platform in the Biomedical Landscape: Chemical, Physical, and Biological Insights
Source: Gels. 2026 Apr 22;12(5):351. doi: 10.3390/gels12050351 (PMC13205765; doi:10.3390/gels12050351)
Supplement: Supplementary file 1 [file gels-12-00351-s001.zip › Supplementaryfile_S2_gels.pdf]

Supplementary file S2:
Detailed protocol for fibrin gel synthesis

1. Reagents

• Distilled and deionized water, **MilliQ-H_2O**.

• **Sodium chloride**, NaCl, CARLO ERBA Reagents (via Raffaele Merendi, 22 20010 Cornaredo (MI) - Italy) product ID n° A194090010.

• **Fibrinogen**, Fraction I, type I-S from bovine plasma, Sigma Aldrich (3050 Spruce Street, St. Louis, MO 63103 - United States of America) product ID n° F8630-10G.

• **Thrombin** from bovine plasma, Sigma Aldrich (400 Summit Drive, Burlington, MA 01803 - United States of America) product ID n° 1.12374.0001.

• **Calcium chloride**, CaCl2, Sigma Aldrich (3050 Spruce Street, St. Louis, MO 63103 - United States of America) product ID n° C4901-500G.

• **Cell culture medium**:

- **α-MEM** w/o L-Glutamine, w/o ribonucleosides, & w/o deoxyribonucleosides, Biowest-the serum specialist (2 rue du Vieux Bourg 49340 Nuaillé - France) product ID n° L0476-500.
- **D-MEM** w 1 g/L glucose, w/o L-Glutamine, w sodium pyruvate, Biowest-the serum specialist (2 rue du Vieux Bourg 49340 Nuaillé - France) product ID n° L0064-500.
- **HAM'S F12** w/o L-Glutamine, EuroClone (Via Figino 20/22, 20016 Pero (MI) - Italy) product ID n° ECB7502L.
- **RPMI 1640** w/o L-Glutamine, Biowest-the serum specialist (2 rue du Vieux Bourg 49340 Nuaillé - France) product ID n° L0501-500.

• **Fetal bovine serum**, FBS, Sigma Aldrich (3050 Spruce Street, St. Louis, MO 63103 - United States of America) product ID n° 12103C-500ML.

• **Penicillin-Streptomycin**, Sigma Aldrich (3050 Spruce Street, St. Louis, MO 63103 - United States of America) product ID n° P4333-100ML.

• **L-Glutamine** 200mM in 0.85% of NaCl solution, Lonza (9900 Medical Center Drive Rockville, MD 20850 - United States of America) product ID n° BE17-605E.

2. Equipment

• Measuring cylinders of different sizes.

• Micropipettes with filtered plastic tips of different volumes.

• Pipette container and pipettes of different volumes.

• Iron spatula.

• Analytical balance.

• Test tubes with volume of 50 mL and 15 mL.

• Microtubes with volume of 1.5 mL and 2 mL.

• Glass bottles with various volumes.

• Becher with various volumes.

• Syringe and syringe filter with 0.22 μm pore size.

• Autoclave.

• Petri dishes with various diameters.

• Multi-well plates.

• Fridge at 4°C.

• Freezer at -20°C.

• Water bath at 37°C.

• Incubator at 37°C.

• Centrifuge.

3. Physiological Solution Preparation

The protocol to prepare a physiological solution is reported:

1. Weigh, using analytical balance and iron spatula, the mass of sodium chloride, aCl, necessary to prepare a 0.9% m/V solution.
2. Transfer the salt inside a Becher.
3. Add, inside the Becher, a certain volume of MilliQ-H_2O, lower than that necessary to reach the desired concentration using a micropipette or pipette.
4. Wait for the complete dissolution of the salt in the solvent.
5. Transfer the solution inside the measuring cylinder.
6. Dilute to the mark with MilliQ-H_2O, to reach 0.9% m/V concentration.
7. Transfer the solution to either a glass bottle or a test tube.
8. Sterilize the solution either by syringe filtration using a filter with 0.22 μm pore size or in the autoclave.

<u>The physiological solution stored at room temperature in sterile conditions can be maintained for at least 6 months.</u>

4. Calcium Chloride Solution Preparation

The protocol to prepare $CaCl_2$ solution is reported:

1. Weigh, using an analytical balance and an iron spatula, the mass of calcium chloride powder ($CaCl_2$) necessary to prepare a solution of 50mM.
2. Transfer the salt inside a Becher.
3. Add, inside the Becher, a certain volume of MilliQ-H2O, lower than that necessary to reach the desired concentration, using a micropipette or pipette.
4. Wait for the complete dissolution of the salt in the solvent.

5. Transfer the solution inside the measuring cylinder.
6. Dilute to the mark with MilliQ-H_2O, to reach 50 mM concentration.
7. Transfer the solution into a test tube.
8. Sterilize the solution either by syringe filtration using a filter with 0.22 μm pore size or in an autoclave.

The calcium chloride solution stored in fridge at 4°C in sterile condition can be maintained for at least 6 months.

5. Fibrinogen Solution Preparation

The protocol to prepare fibrinogen solution is reported:

1. Remove fibrinogen powder from the freezer at -20°C.

NOTE THAT: Fibrinogen powder is very sensitive, and repeated thawing and re-freezing can cause its deterioration. It is suggested to divide the powder into different test tubes to reduce the number of thawing and refreezing.

2. Weigh, using an analytical balance and an iron spatula, the mass of fibrinogen powder necessary to prepare a solution with a concentration that matches the physiological range.

NOTE THAT: Weigh the powder directly inside a test tube because fibrinogen is very electrostatic; avoid the use of boats for weighing

3. Centrifuge for around 30 seconds to facilitate the deposition of fibrinogen powder on the test tube bottom.
4. Add, using a micropipette or pipette, the respective volume of physiological solution to prepare a 2x solution with respect to the physiological range.

NOTE THAT: Not all the fibrinogen powder may be deposited on the test tube bottom after centrifugation. By sliding the liquid along the walls of the test tube, the residue may end up on the test tube bottom, facilitating the dissolution.

5. Move the test tube into the water bath at 37°C.
6. Every 20/30 minutes, gently finger hit the test tube.
7. Wait for the complete dissolution of the powder in the solvent.

NOTE THAT: The complete dissolution usually requires around 2 hours. Do not leave the test tube for more than 3 hours in the bath.

8. Remove the test tube from the water bath at 37°C.
9. Sterilize the solution by syringe filtration using a filter with a 0.22 μm pore size.

NOTE THAT Fibrinogen solution cannot be sterilized in an autoclave.

10. Transfer the solution to the fridge at 4°C.

The fibrinogen solution stored in a fridge at 4°C can be maintained for a maximum of 1 week.

6. Thrombin Solution Preparation

The protocol to prepare the thrombin solution is reported:

1. Remove thrombin powder from the fridge at 4°C.
2. Weigh, using an analytical balance and an iron spatula, the mass of thrombin powder necessary to prepare a solution with an initial concentration of 100 U/mL.

NOTE THAT: It is the proper concentration of a solution for being well-preserved in a freezer at -20°C.

3. Add, in the test tube using a micropipette or pipette, the respective volume of MilliQ-H_2O to reach a 100 U/mL concentration.
4. Wait for the complete dissolution of thrombin in the solvent.
5. Sterilize the solution by syringe filtration using a filter with a 0.22 μm pore size.
6. Aliquot, using a micropipette, the thrombin solution in microtubes.
7. Frozen the aliquots in a freezer at -20°C.
8. Thaw aliquots one by one.

NOTE THAT: Each aliquot can be frozen and thawed around 10 times.

NOTE THAT: Thrombin is extremely lightweight, and the mass required to prepare a concentration of 100 U/mL is extremely low, the preparation of aliquots is recommended to avoid thrombin powder waste.

<u>The aliquots of thrombin solution stored in a freezer at -20°C can be maintained for months, and thawed and refrozen aliquots can be employed around 10 times.</u>

7. Fibrin Gel Preparation

The protocol to synthesize fibrin gel is reported:

1. Thaw the thrombin aliquot, prepared in Section 6.
2. Move the appropriate volume of thrombin in a microtube to reach a 40x concentration with respect to the desired final concentration.
3. Add, using a micropipette, a volume equal to the diluted thrombin of calcium chloride solution, prepared in Section 4, to decrease the concentration by one-half.
4. Dilute, using a micropipette or pipette, the thrombin-$CaCl_2$ solution with cell culture medium in a 1:10 ratio.

NOTE THAT: Both cell culture medium alone and cell culture medium containing FBS, glutamine, and antibiotics such as Penicillin and Streptomycin can be employed.

5. Set aside the microtube.

6. Move a volume of fibrinogen solution, prepared in Section 5, in Petri dishes or multi-wells, using a micropipette, considering that the final concentration of fibrinogen inside the fibrin gel has to match the final concentration selected.
7. Add, in the same Petri dish or multi-well plate, a volume of thrombin plus calcium chloride solution, using a micropipette, so that the final concentration of calcium chloride matches the desired final concentrations.

NOTE THAT: The mixing ratio of the solutions, used merely for convenience, is 9:11 as follows (fibrinogen solution): (thrombin-$CaCl_2$ solution)

8. Move the Petri dish or multi-well plate in the incubator at 37°C.
9. Leave the fibrin gel inside the incubator at 37°C without moving it for a time between a couple of hours and a day.
10. Add either PBS (Phosphate Buffer Solution) or cell culture medium on the surface of fibrin gel to keep it hydrated.

NOTE THAT: It is better to change the liquid on the surface of fibrin gel, using a micropipette, once a week.
